# The effect of family structure on physical activity levels among children and adolescents in Western China in the era of COVID-19

**DOI:** 10.1186/s12889-022-14432-x

**Published:** 2022-11-14

**Authors:** Mengyao Shi, Yuqing Shi, Zuhang Zhao, Xiangyu Zhai, Xiang Fan

**Affiliations:** 1 Department of Physical Education, Shanghai Jiao Tong University, Shanghai, 200240 China; 2 The High School Affiliated to Southern, University of Science and Technology, Shenzhen, 518109 China; 3 Graduate School of Sport Sciences, Waseda University, Saitama, 359-1192 Japan; 4 Shanghai Research Center for Physical Fitness and Health of Children and Adolescents, Shanghai University of Sport, Shanghai, 200438 China

**Keywords:** Physical activity, Children and adolescents, Parental influence, Normalization of COVID-19, Western China

## Abstract

**Purpose:**

This study aimed to examine the levels of physical activity (PA) among children and adolescents in western China, and the influence of parents on their PA, in the era of coronavirus disease 2019 (COVID-19).

**Methods:**

This cross-sectional study used a multistage questionnaire to evaluate 4800 children and adolescents of grades 4–12 (9–17 years old) from 48 primary and secondary schools across 16 districts and counties in western China. In addition to PA, questionnaires collected data on demography, family structure, and exercise habits. Data were assessed using the International Physical Activity Questionnaire-Short Form (IPAQ-SF) and analyzed using chi-square tests, t-tests, Spearman’s correlation, and logistic regression models in SPSS.

**Results:**

In this study, a minority (42.1%, *n* = 1553) of children and adolescents met the daily exercise target (60 min) recommended by the World Health Organization (WHO). Moderate- to vigorous-intensity physical activity (MVPA) level of boys was significantly higher than that of girls. Regardless of sex, children with the highest MVPA levels were those in grades 4–6, and PA levels decreased with increasing age. Furthermore, for every hour of increase in the daily MVPA of parents, the MVPA also increased by 6.1–13.9 min in children and adolescents. Moreover, areas of higher economic development were associated with lower levels of MVPA.

**Conclusions:**

Overall, this study found a low level of MVPA in children and adolescents of western China; both family structure and parental activity have a significant influence on the children's PA levels. Besides, the impact of COVID-19 on PA levels has not been entirely negative.

## Implications and Contribution

Most children and adolescents in western China did not meet the World Health Organization’s recommended daily exercise limit. Boys had higher levels of physical activity, and, for both sexes, exercise levels decreased with increasing age. Parental activities, economic development, and family structure played a role in physical activity levels.

## Background

A significant association exists between healthy exercise habits in children and adolescents and fitness later in life [1]. Worldwide, physical inactivity is the fourth leading cause of death [2], and adequate exercise reduces the risk of obesity and chronic diseases [3]. However, there is a general lack of physical activity (PA) among children and adolescents in China. Indeed, a study conducted in 2017 showed that only 29.9% of children and adolescents living in China met the PA recommendations set by the World Health Organization (WHO) [4]. A meta-analysis conducted in 2020 showed that the average time of moderate- to vigorous-intensity physical activity (MVPA) for Chinese children and adolescents was 37.6 min per day [5], which is much lower than the recommended time of 60 min proposed by the WHO.

Factors affecting PA levels in children and adolescents differ across countries and even among regional sub-populations [6, 7]. Overall, previous studies in China reported low PA levels in children and adolescents, and family factors had a major impact on this [8]. While studies in eastern China have shown low levels of exercise [9], no such research has been carried out in the west. Western China covers an area of 6,781,600 km^2^, accounting for 72% of China’s total territory and 27.2% of the Chinese population. The gross domestic product (GDP) of western China accounts for 20.7% of the country’s total GDP [10]. In terms of economy, western China is less developed than the east, and obesity and other chronic diseases are increasingly affecting younger populations [11].

The impact of family environment in shaping the PA levels of children and adolescents is crucial. Podstawski et al. [12] showed a significant correlation between parental education and their children’s PA level. A systematic review concluded that social factors, such as parental support and income, had an impact on PA levels among children and adolescents [13]. Due to an unbalanced level of social and economic development, family economic environment in western China is different from that in other regions. Few studies have examined PA levels in children and adolescents in western China as well as on the associated family factors. According to government policy, the time and frequency of outdoor activities for Chinese children and teenagers are restricted, and instead, some organized and separate indoor sports and online exercises are given. Therefore, in this study, we aimed to comprehensively investigate the PA status and its influencing factors in children and adolescents during COVID-19 and provide a theoretical basis for formulating PA intervention policies for this population.

## Methods

### Participants

This study included 4800 children and adolescents aged 9–18 years (school grades 4–12) from 48 schools across 16 districts and counties in western China. A cross-sectional multistage sampling method was used to assess PA levels and other sociological factors.

The sampling process was divided into three stages. In the first stage, four local administrative cities were selected from western China. Given the expected economic differences between urban and rural areas, two towns from each rural area and two districts from each city were randomly selected. For the second stage of sampling selection, one primary, one junior high, and one high school were randomly selected within each sampling layer (town or district). The third stage involved the random selection of 100 students from two to four classes from each grade in each sample school. In this study, over 450 students were selected from each grade from grades 4–12. According to GDP per capita, the level of economic development in western China was classified as high (> 80,000 CNY), medium (40,000–80,000 CNY), and low (< 40,000 CNY) [14]

The questionnaires were completed from March 2021 to September 2021. The sampling process was affected by the COVID-19 pandemic and was divided into three phases: before the spring semester, after the spring semester, and before the fall semester. The Chinese government restricted outdoor activities and social activities during this period. In response, all schools in China adopted different policies on student activities. In total, 4800 questionnaires were sent out, of which 4209 were returned and 3697 were considered valid, with an effective rate of 87.8%. Of the 512 exclusions, 293 questionnaires were removed due to missingness, and 219 due to abnormal data.

The questionnaire was approved by the principals and teachers at participating schools. Written informed consent was obtained by participants, participation was voluntary, and all data were anonymized. Before filling in the questionnaire, the participants and their parents were informed about the purpose of the study, the method of filling in the questionnaire, the research process, and the possible inconvenience.

### Physical activity

The PA levels of the participants and their parents were assessed using the International Physical Activity Questionnaire-Short Form (IPAQ-SF), which has been widely used in children, adolescents, and adults in China [15, 16]. The reliability and validity of the scale have been previously verified in the relevant population (Cronbach’s α: 0.79) [17]. The IPAQ-SF included questions about the frequency and duration of vigorous and moderate-intensity PA, walking, and sedentary behavior (SB) over the past 7 days. In this study, the PA of children and adolescents was reported by the participants themselves, while their parents self-reported their PA levels. The investigators explained the definition of PA, and a special emphasis was placed on sports that include indoor exercise, resistance training, and online interaction during the COVID-19 pandemic.

According to recommendations set by the WHO, the average daily time of MVPA should be at least 60 min for children and adolescents (5–17 years old) [18]. Therefore, participants who reached the recommended level were assigned to the sufficient PA group, and others to the insufficient PA group.

### Sedentary behavior time in parents

Parents’ low-energy waking behavior, such as sitting or lying down during working hours or leisure time, was defined as SB. Previous studies assessed SB by self-reporting [19]. Therefore, parents of participants were asked to report their daily sedentary time for a week, including sitting at work and leisure, lying or sitting while chatting, reading, using cell phones, computers, or watching television.

### Family demographics

In the questionnaire, participants’ parents answered questions on the number of children in the household and living arrangements. Implementation of the family planning policy in the northwest differed from that in the east, which created a difference in family structure. The number of children in a household was divided into two categories: 1) one child, 2) more than 1 children. The living arrangement was classified into three categories: 1) living with both parents, 2) living with parents and grandparent(s), 3) living with one parent only.

### Statistical analyses

Mean values ± standard deviation ($\overline{x}$±SD) were used to describe the continuous variables with normal distribution. Non-normally distributed data were calculated the median and IQRs. The chi-squared test or t-test was used to compare differences in demographic variables and the Mann–Whitney U test was used to compare data with non-normal distributions between groups. The Spearman correlation test was used to verify the correlation between continuous variables with non-normal distributions. Logistic regression analysis with odds ratios (ORs) and 95% confidence intervals (CIs) were used to explore the effects of different factors on adherence to the PA recommendations. The effect of parental activity on the PA levels of participants was analyzed using a linear regression model. *P* < 0.05 was considered statistically significant. All data were analyzed using SPSS version 25.0 (International Business Machines).

## Results

### Participant characteristics

The sample included 1293 primary school students (grades 4–6, 35.0%), 1209 junior high schools (grades 7–9, 32.7%), and 1195 high school students (grades 10–12, 33.2%). The mean age of the participants was 12.9 ± 2.6 years. In total, 986 participants were included in the high-level, 916 in the middle-level, and 1795 in the low-level areas (Table 1).

**Table 1:** Demographic characteristics of participants

Variable	Overall (*n* = 3697)	Boys (*n* = 1805)	Girls (*n* = 1892)	*P*-value
	n (%)or mean ± SD or medians (IQRs)			
Age in years	12.9 ± 2.6	13.0 ± 2.6	12.9 ± 2.6	0.76
Grade				0.66
4–6	1293 (35.0%)	628 (34.8%)	664 (35.1%)	
7–9	1209 (32.7%)	581 (32.2%)	628 (33.2%)	
10–12	1195 (32.3%)	596 (33.0%)	599 (31.7%)	
Residential location^1^				0.49
Urban	2049 (55.4%)	994 (55.1%)	1055 (55.8%)	
Rural	1555 (42.1%)	760 (42.1%)	794 (42.0%)	
Economic development				0.95
High	986 (26.7%)	486 (27.0%)	500 (26.5%)	
Moderate	916 (24.8%)	446 (24.7%)	470 (24.9%)	
Low	1795 (48.5%)	873 (48.3%)	922 (48.7%)	
Number of children^2^				< 0.01
one	1402 (37.9%)	749 (41.5%)	652 (34.5%)	
more	2284 (61.8%)	1051 (58.2%)	1233 (65.2%)	
Living with^3^				0.41
Both parents	2917 (78.9%)	1441 (79.8%)	1475 (78.0%)	
Grandparent(s)	520 (14.1%)	250 (13.9%)	270 (14.3%)	
One parent	235 (6.4%)	105 (5.8%)	130 (6.9%)	
PA level				< 0.01
sufficient	1553 (42.1%)	809 (44.9%)	744 (39.4%)	
insufficient	2144 (57.9%)	996 (55.1%)	1148 (60.6%)	
Students’ MVPA^4^	42.9 (21.4–77.1)	45.7 (23.8–81.4)	39.3 (20.0–74.1)	< 0.01
Parents’ MVPA^4^	30.0 (4.3–77.1)	28.6 (2.1–77.1)	30.0 (5.7–75.7)	0.36
Parents’ SB^4^	120.0 (8.6–231.4)	114.3 (0–228.6)	120.0 (15.5–235.7)	0.38

*PA* physical activity, *MVPA* moderate- to vigorous-intensity physical activity, *SB* sedentary behavior, *SD* standard deviation, *IQR* interquartile range

^1^93 missing datapoints

^2^11 missing datapoints

^3^25 missing datapoints

^4^medians (IQRs) for Non-normally distributed data; unit: minutes/per day

### Moderate- to vigorous-intensity physical activity in participants

About 42.1% of children and adolescents living in western China had reached sufficient levels of MVPA, and the proportion of boys (44.9%) was significantly higher than that of girls (39.4%) (*p* < 0.01) (Table 2). Regardless of sex, MVPA was progressively reduced with the increase in school grade. Further, there were significant differences in MVPA levels of children and adolescents based on different levels of economic development. Participants living in areas of high economic development had the lowest levels of MVPA. Only 34.0% of students who lived in a one-child family had a significantly lower percentage in sufficient MVPA than those living in a multiple-child family.

**Table 2:** Moderate- to vigorous-intensity physical activity (MVPA) of participants under different sociological variable

Variable	MVPA		t/F	χ^2^
	Time(Mean ± SD)	sufficient(n /rate)		
Sex			4.301^**^	11.383^**^
Boy	69.02 ± 59.43	809(44.9%)		
Girl	60.83 ± 56.28	744(39.4%)		
Grade			52.013^**^	73.892^**^
4–6	77.91 ± 65.12	666(51.6%)		
7–9	58.22 ± 52.98	447(37.0%)		
10–12	57.36 ± 51.87	440(36.9%)		
Residential location			0.754	2.333
Urban	65.62 ± 59.51	868(42.4%)		
Rural	64.14 ± 56.10	653(42.1%)		
Economic development			42.090^**^	56.824^**^
High	53.85 ± 50.65	335(34.0%)		
Moderate	59.74 ± 53.69	357(39.0%)		
Low	73.49 ± 62.40	861(48.1%)		
Number of children			-1.072	4.696^*^
one	63.51 ± 58.73	558(39.9%)		
more	65.70 ± 57.61	992(43.5%)		
Living with			2.148	5.384
Both parents	65.96 ± 59.15	1253(43.0%)		
Grandparent(s)	62.20 ± 56.08	203(39.1%)		
One parent	57.03 ± 46.20	87(37.0%)		
PA level			60.002^**^	
sufficient	115.84 ± 56.30	3697(100%)		—
insufficient	27.79 ± 15.46	——		

^*^P < 0.05

^**^P < 0.01. Unit for minute/per day. PA: physical activity

With increase in age, the time of MVPA in children and adolescents showed a significant downward trend in both genders. (Table 3). Significant linear positive correlations were found between the MVPA of participants and the MVPA of parents. The parents’ SB and the MVPA of participants showed different correlations between the sexes.

**Table 3:** Correlation between different variables and moderate- to vigorous-intensity physical activity (MVPA) of participants

Variable	Boys		Girls	
	β	*P*-value	β	*P*-value
Age in years	-0.14^**^	< 0.01	-0.17^**^	< 0.01
parents’ MVPA^2^	0.11^**^	< 0.01	0.12^**^	< 0.01
parents’ SB^2^	0.07^**^	< 0.01	-0.05^*^	0.04

^2^Unit for minute/per day

^*^P < 0.05

^**^P < 0.01. *MVPA* moderate- to vigorous-intensity physical activity, *SB* sedentary behavior time

### *Differences in adherence to moderate- to vigorous-intensity physical activity* recommendations

Table 4 displays the differences in adherence to MVPA recommendations based on family structure. In school grades 10–12, a significant correlation was noted between the number of children in household and PA levels even after adjustment for age, BMI, residential location, and economic development. This correlation was especially strong in girls. After BMI is further included as each other's confounding factors, the aORs (adjusted odds ratios) for reporting high levels of PA still remained strong.

**Table 4:** Adjusted odds ratios (with 95% confidence intervals) of meeting physical activity recommendation by family structure

Family structure		Grade 4–6		Grade 7–9		Grade 10–12	
		Boys	Girls	Boys	Girls	Boys	Girls
Model 1	Number of children						
	One	1.000 (Ref.)	1.000 (Ref.)	1.000 (Ref.)	1.000 (Ref.)	1.000 (Ref.)	1.000 (Ref.)
	More	1.006 (0.724–1.399)	0.994 (0.711–1.391)	0.715 (0.490–1.042)	0.924 (0.621–1.373)	1.558^*^ (1.058–2.292)	1.925^**^(1.209–3.063)
	Living with						
	Both parents	1.000 (Ref.)	1.000 (Ref.)	1.000 (Ref.)	1.000 (Ref.)	1.000 (Ref.)	1.000 (Ref.)
	Grandparent(s)	0.863 (0.560–1.332)	0.888 (0.589–1.340)	0.640 (0.376–1.089)	0.542^*^(0.326–0.902)	0.990 (0.607–1.615)	1.104 (0.646–1.884)
	One parent	0.377^*^(0.167–0.849)	0.806 (0.409–1.591)	0.746 (0.392–1.421)	0.735 (0.401–1.346)	1.276 (0.617–2.640)	1.301 (0.670–2.528)
Model 2	Number of children						
	One	1.000 (Ref.)	1.000 (Ref.)	1.000 (Ref.)	1.000 (Ref.)	1.000 (Ref.)	1.000 (Ref.)
	More	1.025 (0.740–1.421)	0.966 (0.700–1.335)	0.826 (0.581–1.173)	1.141 (0.783–1.663)	1.477^*^ (1.046–2.084)	1.805^**^(1.196–2.724)
	Living with						
	Both parents	1.000 (Ref.)	1.000 (Ref.)	1.000 (Ref.)	1.000 (Ref.)	1.000 (Ref.)	1.000 (Ref.)
	Grandparent(s)	1.012 (0.643–1.591)	1.049 (0.685–1.606)	0.708 (0.423–1.182)	0.563^*^(0.331–0.957)	1.112 (0.685–1.806)	0.940 (0.549–1.610)
	One parent	0.550 (0.256–1.182)	1.245 (0.628–2.470)	0.782 (0.419–1.459)	0.839 (0.462–1.521)	1.018 (0.501–2.069)	1.359 (0.706–2.618)

Note: ^*^P < 0.05, ^**^P < 0.01

Model 1: adjusted for age, residential location, and economic development; Model 2: adjusted for BMI, age, residential location, and economic development

In grades 7–9, the MVPA of girls living with their grandparent(s) was significantly lower than that of girls who living with both their parents (aOR = 0.542; 95% CI = 0.326–0.902).

In grades 4–6, the MVPA of boys living with a single parent was only 0.377 ( 95% CI = 0.167–0.849) times that of the boys living with both parents. After further including BMI as confounding factors, the aOR for reporting high levels of PA among grade 7–9 girls (aOR = 0.563; 95% CI = 0.331–0.957) still remained significant difference.

### Relationship between parents’ behavior and MVPA of participants

We assessed the parents' PA and sedentary behaviors. In all subgroups, except boys in grades 4–6, significant positive correlations were found between the parents’ and participants’ MVPA, which remained even after adjustment for age, BMI, residential location, and economic development. For every 1-h increase in the daily MVPA of the parents, the MVPA among children and adolescents also increased by 6.1–13.9 min. This positive correlation was the strongest for girls in grades 7–9. However, no significant correlation between children's PA and Parents' SB was observed after further adjustment for possible confounding factors. (Table 5).

**Table 5:** Odds ratios (with 95% confidence intervals) of parental moderate to vigorous physical activity (MVPA) and sedentary behavior

Variable	Parents’ MVPA		Parents’ SB	
	β(95% CI)	*P*-value	β(95% CI)	*P*-value
Boys				
Grade 4–6	0.032 (-0.014–0.079)	0.163	-0.010 (-0.027–0.035)	0.371
Grade 7–9	0.136^**^ (0.032–0.240)	0.008	0.016 (-0.008–0.040)	0.188
Grade 10–12	0.145^**^ (0.013–0.277)	0.006	0.008 (-0.015–0.030)	0.516
Girls				
Grade 4–6	0.091^**^ (0.032–0.150)	0.003	-0.002 (-0.024–0.021)	0.892
Grade 7–9	0.232^**^ (0.148–0.309)	0.000	-0.017(-0.037–-0.003)	0.029
Grade 10–12	0.019 (0.015–0.053)	0.276	-0.070 (-0.150–0.010)	0.087

Note: adjusted for age, BMI, residential location and economic development

^*^P < 0.0

^**^P < 0.01

## Discussion

Overall, this study found a low level of MVPA among children and adolescents in western China. Only 42.1% of the participants had sufficient MVPA levels. Boys engaged in PA for longer and were more likely to reach sufficient levels of PA compared to girls. Regardless of sex, children with the highest MVPA levels were those in grades 4–6. With increasing grades and the intensification of academic tasks and pressure, the MVPA levels gradually decreased. This conclusion was in agreement with those reached by previous studies [20]. To improve health and boost the PA levels of children and adolescents, the Chinese government issued a new policy on development, including PA, in 2021.

However, due to the Chinese government's policy of limiting outdoor activities during the COVID-19 pandemic, people’s exercising habits have been disrupted. Consequently, PA levels of children and adolescents decreased significantly during the pandemic [21, 22]. However, PA levels after 2020, when COVID-19 had become “normalized,” remained unclear. We found that the impact of COVID-19 on PA among children and adolescents was not entirely negative as all Chinese primary and secondary schools had adopted new ways to ensure students maintained healthy levels of PA while on campus—Webcast course. In addition, mobile applications using artificial intelligence were developed that could recognize human movements and provide rankings so that students could participate in online sports games. A previous study showed that the MVPA level of children and adolescents in the post-epidemic era has increased significantly compared with that during the pre-epidemic period [23]. Particularly, vital capacity, flexibility, and muscular strength were significantly improved during the COVID-19 pandemic lockdown periods [24].

Previous studies have shown that PA levels of children and adolescents are significantly correlated with the economic development where they live [25]. In this study, we showed that higher economic development is correlated with lower levels of MVPA among children and adolescents living in China, which is consistent with the findings of previous global studies [6, 26]. Contrarily, a study by Wang et al. [27] conducted in China concluded that residents in economically developed areas participated in more PA than did the residents who lived in economically underdeveloped areas. This discrepancy may be attributed to the overall economic development in China and its individual regions. Previous studies have shown an opposing relationship between economic development and PA levels in developed and developing countries [27].

This study showed that high school students with siblings spent significantly more time participating in MVPA than do those without siblings; this may be due to increasing academic pressure they experience when they enter high school [28]. Adolescents had decreased PA levels in the family, which, however, increased in the company of peers. Indeed, Beets et al. [29] showed that the companionship of peers significantly increased the level of PA of children and adolescents. Previous studies have demonstrated that as children and adolescents enter puberty, they become more dependent on and spend more time with their peers [30, 31]. At the same time, adolescents in multi-child families begin to share some of the responsibilities of caring for younger siblings and housework as they grow older, which could explain the observed increase in MVPA.

In addition, associations between children's PA and who they live with have been reported before. Previous studies that investigated MVPA among adolescents in Shanghai in 2017 resulted that compared with the participants who lived with both parents, the PA of primary school boys living with one parent and junior high school girls living with their grandparents, was significantly low [9, 32]. The preliminary results of our study indicated that MVPA levels in children and adolescents in two-parent families was generally higher compared with those from other households. Previous studies explained that two-parent families paid more attention to the overall development of children and the positive impact of PA on their health. Parents may also spend more time accompanying their children in PA [33]. In addition, according to a study performed in 2012, children who lived with their grandparents were more likely to have lower levels of PA as did their grandparents. Adolescents had differing interests in sports and PA [34] and grandparents tend to be overprotective when raising their grandchildren [35].

Similarly to previous studies, we found a significant positive correlation between MVPA levels of parents and their children [13, 36]. A Spanish study found significant correlations between parental exercise and children’s PA levels in children aged 6–10 years [37]. A study conducted by Framingham et al. [38] showed that children with sedentary parents were only one-sixth as likely to be physically active as those with active parents. The influence of the behavior of role models, such as parents, is a core concept in social learning theory [39]. Therefore, reducing parental SB and increasing PA would set a good example for children. At the same time, it would have physical and psychological benefits for the parents themselves, including the prevention of chronic diseases such as obesity [40].

This study had some limitations. The MVPA data were self-reported, which might have resulted in biases, such as recall or social desirability bias, leading to the overestimation of PA levels. Objective measurement methods such as an accelerometer can be used in future studies aimed at investigating PA behaviors. Furthermore, the cross-sectional design limited inferences regarding causality.

Despite these limitations, this study provided deeper insight into the PA levels of children and adolescents, and the relationship between parental factors and PA in children and adolescents in western China during the COVID-19 pandemic.

## Conclusion

This study showed that the PA levels of most children and adolescents were insufficient in western China and was affected by family structure, economic development, and parental activity. The COVID-19 pandemic has had an impact on PA levels in this population, but the development of new applications and sports games was a positive outcome. Nevertheless, the influence of family members, especially parents, on children’s PA levels remains crucial in the era of COVID-19.

## Data Availability

The datasets generated and/or analyzed during the current study are not publicly available due to privacy concerns of participants but are available from the corresponding author on reasonable request.

## Abbreviations

COVID-19: Coronavirus disease 2019
CIs: Confidence intervals
IPAQ-SF: International Physical Activity Questionnaire-Short Form
MVPA: Moderate- to vigorous-intensity physical activity
ORs: Odds ratios
PA: Physical activity
SB: Sedentary behavior
WHO: World Health Organization
